# Comparison of Different Machine Learning Models in Prediction of Postirradiation Recurrence in Prostate Carcinoma Patients

**DOI:** 10.1155/2022/7943609

**Published:** 2022-02-07

**Authors:** Mladen Marinkovic, Marina Popovic, Suzana Stojanovic-Rundic, Milos Nikolic, Milena Cavic, Dusica Gavrilovic, Dusan Teodorovic, Nenad Mitrovic, Ljiljana Mijatovic Teodorovic

**Affiliations:** ^1^Department of Radiation Oncology, Clinic for Radiation Oncology and Diagnostics, Institute for Oncology and Radiology of Serbia, Belgrade, Serbia; ^2^Department of Nuclear Medicine, Institute for Oncology and Radiology of Serbia, Belgrade, Serbia; ^3^Faculty of Medicine, University of Belgrade, Belgrade, Serbia; ^4^Faculty of Transport and Traffic Engineering, University of Belgrade, Belgrade, Serbia; ^5^Department of Experimental Oncology, Institute for Oncology and Radiology of Serbia, Belgrade, Serbia; ^6^Data Center, Institute for Oncology and Radiology of Serbia, Belgrade, Serbia; ^7^Serbian Academy of Sciences and Arts, Belgrade, Serbia; ^8^Faculty of Mechanical Engineering, University of Belgrade, Belgrade, Serbia; ^9^Faculty of Medical Sciences, University of Kragujevac, Kragujevac, Serbia

## Abstract

After primary treatment of localized prostate carcinoma (PC), up to a third of patients have disease recurrence. Different predictive models have already been used either for initial stratification of PC patients or to predict disease recurrence. Recently, artificial intelligence has been introduced in the diagnosis and management of PC with a potential to revolutionize this field. The aim of this study was to analyze machine learning (ML) classifiers in order to predict disease progression in the moment of prostate-specific antigen (PSA) elevation during follow-up. The study cohort consisted of 109 PC patients treated with external beam radiotherapy alone or in combination with androgen deprivation therapy. We developed and evaluated the performance of two ML algorithms based on artificial neural networks (ANN) and naïve Bayes (NB). Of all patients, 72.5% was randomly selected for a training set while the remaining patients were used for testing of the models. The presence/absence of disease progression was defined as the output variable. The input variables for models were conducted from the univariate analysis preformed among two groups of patients in the training set. They included two pretreatment variables (UICC stage and Gleason's score risk group) and five posttreatment variables (nadir PSA, time to nadir PSA, PSA doubling time, PSA velocity, and PSA in the moment of disease reevaluation). The area under the receiver operating characteristic curve, sensitivity, specificity, positive predictive value, negative predictive value, and predictive accuracy was calculated to test the models' performance. The results showed that specificity was similar for both models, while NB achieved better sensitivity then ANN (100.0% versus 94.4%). The ANN showed an accuracy of 93.3%, and the matching for NB model was 96.7%. In this study, ML classifiers have shown potential for application in routine clinical practice during follow-up when disease progression was suspected.

## 1. Introduction

Prostate carcinoma (PC) is the second most frequently diagnosed carcinoma and the fifth leading cause of carcinoma-related deaths in the male population worldwide [1]. Approximately 1.4 million new PC cases occurred in 2020 worldwide, with an incidence rate of 4.34 per 100.000 in Serbia [1]. Approximately, 10% of newly diagnosed PC patients is presented with bone metastases and it is increasing to 80% at advanced stages of the disease [2]. Metastases are related to poor prognosis, bone pain, and indicate the incurability of disease in most cases with a 5-year survival rate of 25% and median survival of approximately 40 months [3, 4]. Patients with local or regional disease achieve a 5-year survival greater than 99%, but in patients with distant metastases, the 5-year survival drops to 30% [5].

The decision of optimal treatment for localized PC is based on numerous factors, but the most important are the following: T stage, Gleason's score (GS), and initial serum prostate-specific antigen (PSA) level [6]. European Association of Urology suggested classification of patients with localized PC based on the probability for biochemical recurrence after definitive local treatment [7]. Treatment options for localized PC are the external beam radiation therapy (EBRT) alone or with the addition of androgen deprivation therapy (ADT) based on clinical indications, brachytherapy, radical prostatectomy, or active surveillance.

Posttreatment PSA surveillance has resulted in earlier detection of PC progression. After the treatment of localized PC, approximately up to a third of patients exhibit disease recurrence [8, 9]. When an increase in PSA level is detected during follow-up, it is primarily important to distinguish whether it is caused by locoregional recurrence or metastatic disease, which has a huge impact on further treatment course. By implementation of prostate multiparametric magnetic resonance imaging (mpMRI), the presence of locoregional recurrence can be detected at lower PSA levels [10]. The standard workup to detect metastatic PC usually includes the 99mTc-methylene diphosphonate bone scan (BS) and chest/abdomen/pelvis scan, or if available, the 18F-choline and gallium-68 prostate-specific membrane antigen (PSMA) positron emission tomography-computed tomography (PET/CT) or whole-body MRI [11]. The advantage of the use of PET/CT or whole-body MRI is the possibility to inspect all parts of the body at the same time. Visschere et al. in their systematic review concluded that a combination of mpMRI to assess the presence of local relapse and PET/CT for the detection of distant metastases is the optimal choice in this setting [12].

Different predictive models have already been used either for initial stratification of patients or to predict disease recurrence [13]. In 1993, the Partin tables were the first to predict the pathohistological stage of disease based on PSA level, GS, and clinical stage [14]. However, none of the current models has a high predictive accuracy, and they are mainly focused on the prediction of the recurrence after radical prostatectomy [15, 16]. Recently, artificial intelligence has been introduced in the diagnosis and management of PC with a high potential to revolutionize this field [17, 18].

This study is aimed at developing and comparing two machine learning (ML) classifiers based on artificial neural networks (ANN) and naïve Bayes (NB) in order to predict disease progression in the moment of PSA elevation during the follow-up of PC patients treated with radical radiotherapy with/or without ADT.

## 2. Materials and Methods

### 2.1. Patient Characteristics and Treatment

**Figure 1:**
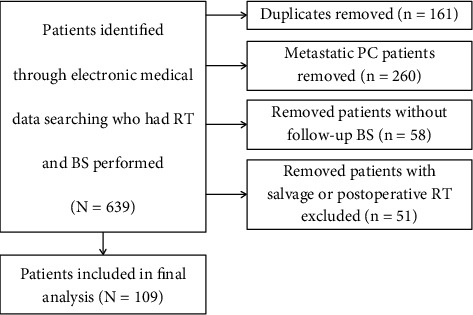
Flowchart of patient selection.

We retrospectively reviewed the medical records of patients treated with radiotherapy for prostate adenocarcinoma between January 2015 and December 2019 at the Institute for Oncology and Radiology of Serbia. We used the performed BS as the criteria in medical database search in order to identify patients with suspicious disease progression during follow-up. The inclusion criteria were as follows: completed radical course of EBRT alone or in combination with ADT, as the primary approach. We excluded patients with metastatic disease, those with performed salvage or postoperative RT and also patients who did not have BS during follow-up. (Figure 1) The final analysis was conducted on 109 patients, which has been shown to meet the criteria of a minimum number of necessary samples of PC in Serbia according to incidence and population size using the 95% confidence level [19].

All patients included in our study initially had either an ultrasound, computed tomography (CT), or MRI of the pelvis. Also, an initial chest radiographs and ultrasound or CT of the abdomen were applied to all patients. For intermediate or high risk category of patients, BS was performed. Patients with metastatic disease at initial work-up were excluded. Due to PSA elevation in the follow-up period, all included patients had at least one disease reevaluation.

The clinical and pathological tumor characteristics at baseline were collected from the institutional electronic medical record database. The clinical data of interest were the patients' information, as well as disease and treatment characteristics, such as the initial PSA level, the GS, and tumor differentiation. The initial staging of the tumor was reevaluated according to the eighth edition of the Union for International Cancer Control (UICC) TNM staging system for PC [20]. Data collection also included information from the follow-up period with a focus on disease recurrence.

Radiation therapy was delivered by an anteroposterior-posteroanterior (2D technique), 3D conformal radiation therapy (3D CRT), or volumetric modulated arc therapy (VMAT) technique. The range of the dose was from 65 to 72 Gy. According to the decision of a multidisciplinary team, for high and intermediate risk patients, ADT was also applied. The ADT included a luteinizing hormone-releasing hormone (LHRH) agonist, alone or in combination with antiandrogen therapy, for a total duration ranging from 3 to 24 months, before, during, and after radiation (neoadjuvant, concurrent, and adjuvant ADT). For some patients with low risk disease and a very large prostate, neoadjuvant ADT was applied in shorter period than 12 months, in order to shrink the prostate and decrease the risk of radiation side effects.

### 2.2. Patient Follow-up

Patients' follow-up was performed every 3 months during the first two years after completion of treatment, and every 6 months thereafter. Clinical examination, PSA level measurement, and ultrasound or CT of abdomen and pelvis were performed at each follow-up visit.

All analyzed patients had PSA elevation during follow-up. At the time of PSA increase, each patient underwent an imaging accompanied by either a CT or MRI of the abdomen and pelvis, a BS, and chest radiographs or CT of the chest, to exclude disease progression. The value of PSA in the moment of disease reevaluation was obtained and used for analysis.

Under no radiological signs of progression, active surveillance was thereby indicated, with control of PSA level in shorter periods. If any progression was detected by imaging diagnostic methods and/or biochemical relapse, a decision on second-line treatment (ADT, chemotherapy, surgical castration, or palliative radiotherapy) was made by a multidisciplinary tumor board. Patients without radiological signs of progression who received second-line treatment were categorized as the biochemical relapse.

The parameters of interest from the period after treatment completion were nadir PSA, time to nadir PSA, PSA doubling time (PSADT), and PSA velocity. The nadir PSA was defined as the lowest PSA value after the end of the RT treatment. Time to nadir PSA was marked as time from the end of RT till nadir PSA is achieved. Calculations of the PSADT and PSA velocity were based on measuring the PSA levels in intervals of at least 3 months. The calculation incorporated the nadir PSA and all subsequent PSA values until the disease reevaluation. The values of PSADT and PSA velocity were performed using the Memorial Sloan-Kettering Medical Center PC prediction tool [21].

### 2.3. Developing of ML Classifiers

Two ML algorithms were applied to discover prediction patterns of progression in PC patients from the available data: artificial neural networks and naïve Bayes Classifier.

The ANNs are information-processing systems capable of learning from experience and able to apply it to new cases with the aim to generalize previous patterns [22]. They are trained to generate an output as a combination between the input variables on the basis of multiple hidden layers. NB classifier is based on the generally known Bayes theorem. The system functions by learning and evaluating the prior probability of belonging to each class using the training data [23]. The main advantage of the implementation of ML models in medicine is the fact that they can detect key features from complex data sets and identify patterns and relationships between them, which cannot be achieved using classical statistical tests.

Patients were randomly divided into two sets. The training set which had 72.5% patients (79 patients) and the testing set which consisted of the remaining patients (30 patients). Patients in both sets were categorized according to the presence/absence of the disease progression into category 0 (patients without disease progression) and category 1 (patients with disease progression). The term disease progression included local recidive, regional and distant lymph node metastases, and bone, liver, and lung metastases, as well as biochemical relapse. When the proportion of patients with disease progression as well as other parameters was considered, the sets were similar.

The training set was used to develop classifiers. The presence/absence of disease progression was defined as the output for developing ML classifiers. The input variables were conducted from the univariate analysis preformed among two groups of patients in the training set. After training the data, the developed models were evaluated using the testing set.

### 2.4. Software and Statistical Analysis

For normal distribution data testing, the Kolmogorov-Smirnov and Shapiro-Wilk tests were used. Descriptive methods (frequencies, percent, mean, median, standard deviation (SD), and range) were used to summarize the data. The statistical significance level was set at *p* < 0.05. For comparison of disease and treatment characteristics among different patient subgroups, the Wilcoxon rank sum, Pearson chi-square, and Fisher exact tests were used. The receiver operating characteristic (ROC) curve methods were applied to investigate the discriminative potential of nadir PSA, time to nadir PSA, PSADT, PSA velocity, and PSA in the moment of disease reevaluation for the presence/absence of disease progression (AUC ROC-area under the ROC curve according to DeLongs method; likelihood ratio test for AUC ROC; the best cut-off value for these parameters was set as value with maximum sensitivity and specificity). The statistical analysis was done with the program R (version 3.3.2 (2016-10-31) —“Sincere Pumpkin Patch”; Copyright (C) 2016. The R Foundation for Statistical Computing; Platform: x86_64-w64-mingw32/×64 (64-bit); downloaded: January 21, 2017). For the artificial neural networks, software Weka 3.8.4 was used while the software for the naïve Bayes classifier was developed in Java programming language (in NetBeans IDE 8.2 development environment).

To select the ML model with the best prediction performance, we evaluated the AUC ROC, sensitivity, specificity, positive predictive value, negative predictive value, and predictive accuracy for each model. AUC ROC curve was used to evaluate how well the model distinguished patients with and without diseases progression. An AUC of 0.5 indicates that the model does not predict better than chance. The discrimination of a diagnostic model is considered perfect if AUC is equal to 1, good if AUC is greater than 0.8, moderate if AUC is 0.6-0.8, and poor if AUC is lesser than 0.6 [24]. ROC curves were compared using pairwise testing.

## 3. Results

Patients', disease, treatment, and follow-up characteristics for both groups of patients, as well as the comparison between groups, are presented in Table 1. The majority of patients had T2-T3 stadium, GS of 7, and N0 disease. Almost two third of patients was treated with radiotherapy combined with ADT, and seventy-two patients developed disease recurrence. A similar percentage of patients had disease progression in both groups (68.4% vs. 60%).

In order to find suitable parameters for developing ML models, we compared patients with and without disease progression in the moment of PSA increase during follow-up in the training group. Comparison of characteristics among patients with and without disease progression in the training group is presented in Table 2. Patients with disease recurrence had a higher baseline PSA level but statistical significance was not confirmed (*p* = 0.124). Patients with disease progression had a significantly higher mean nadir PSA levels (*p* = 0.04) and shorter median time to nadir PSA (*p* < 0.01) compared to patients without progression. Patients who developed disease progression had a lower PSADT and a higher PSA velocity (*p* < 0.01). In our data set, there was no significant correlation between T stage and the risk of disease progression, but a positive trend was found (*p* = 0.05). The only pretreatment parameter that was significantly different among the compared groups was the GS (*p* < 0.01).

**Figure 2:**
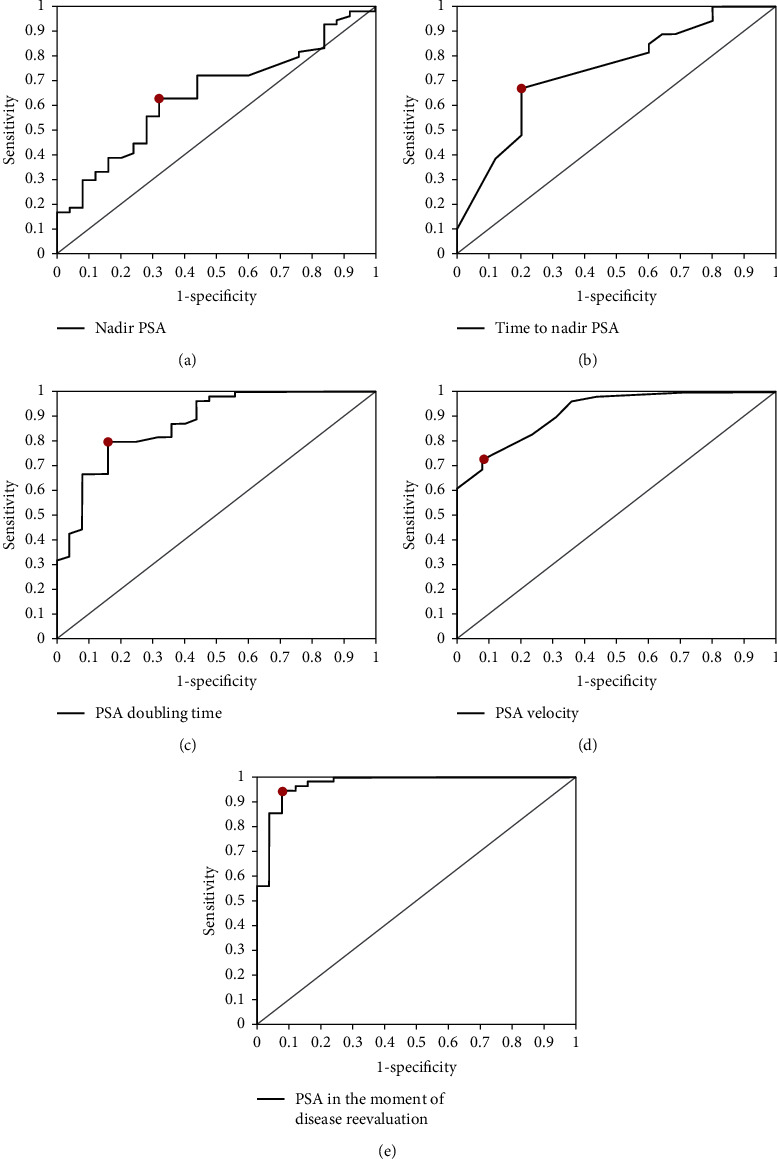
ROC curves for the nadir PSA (a), time to nadir PSA (b), PSA doubling time (c), PSA velocity (d), and PSA in the moment of disease reevaluation (e) in relation to disease progression during follow-up.

The ROC revealed optimal cut-off values for nadir PSA, time to nadir PSA, PSADT, PSA velocity, and PSA in the moment of disease reevaluation, above/below which the risk of the presence of disease progression increased significantly (Table 3 and Figure 2). Next, we examined if there were differences between patients with and without disease progression according to the cut-off values obtained by ROC (Table 4). ROC analysis revealed an optimal cut-off value for nadir PSA of 0.3095 ng/mL, with a significant increase of the risk of progression in patients having a nadir PSA ≥ 0.3095 ng/mL (*p* = 0.01).

Significant variables from the univariate analyses were then used for the construction of ML models. We also included the UICC stage where a positive trend was found. All variables were categorical. Finally, the input variables were two pretreatment parameters: UICC stage (1 = stage I and II; 2 = stage III and IV) and GS risk group (1 = GS6; 2 = GS7; 3 = GS8 − 10), and five variables from the period after treatment completion: nadir PSA (1 for ≤0.3095; 2 for >0.3095), time to nadir PSA (1 for ≤6.5; 2 for >6.5), PSADT (1 for ≤5.05; 2 for >5.05), PSA velocity (1 for ≤0.55; 2 for >0.55), and PSA in the moment of disease reevaluation (1 for ≤6.49; 2 for >6.49). The dependent variable was defined as the presence (category 1) or absence (category 0) of the disease progression. The training group of patients was presented to the ML models during the training, and the network was adjusted according to its error. The second set, composed of 30 patients, was used for testing. The ML outputs were then compared with the known endpoint from the medical records. These patients' characteristics had no effect on training and so provided an independent measure of network performance during and after training. The same sets of patients were used when we tested the ANN and NB approaches.

**Figure 3:**
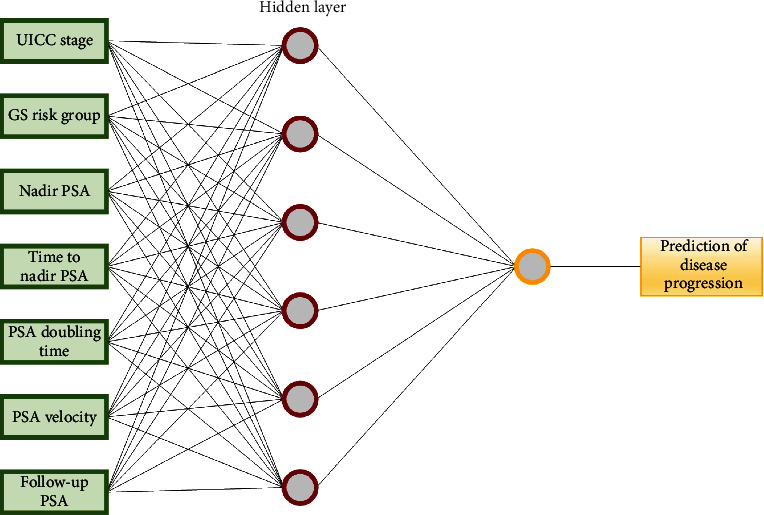
The artificial neural network architecture for predicting presence versus absence of disease progression.

Different configurations of the artificial neural networks were explored. The best result was obtained for the artificial neural network with one hidden layer with 6 neurons and 500 epochs for the training time parameter (Figure 3).

**Figure 4:**
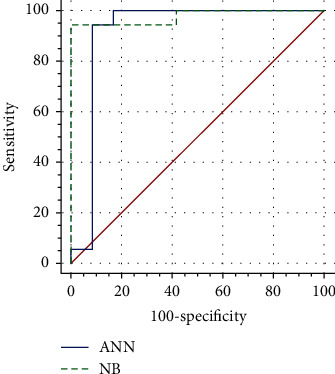
ROC curves of ANN and NB model for prediction of disease progression.

The ROC curves of both models are presented in Figure 4.

Both models exhibited satisfactory predictive characteristics, but the NB model was more successful, with 29 identical results to the data from medical history (96.7% of cases). The matching for ANN model was 93.3% (28 correctly classified patients). Sensitivity, specificity, positive predictive value, negative predictive value and predictive accuracy for proposed classifiers, and comparison between models are presented in Table 5. The ANN model achieved AUC of 0.92, and on the other hand, the NB model reached higher AUC value (0.98). When these two models were compared, no statistical difference was found (*p* = 0.4402).

To obtain the importance of inputs, we applied the method proposed by Garson [25] (we followed the example given in the paper [26]). It uses the final ANN weights between the neurons. We applied the method to our artificial neural network, and we get the results given in Table 6. According to the obtained results, the most significant inputs are Inputs 1, 7, and 2. The lowest value of relative importance has Input 4.

## 4. Discussion

The present study aimed to compare the performance of ML algorithms to stratify patients with PC in the moment of PSA increase during follow-up into low- or high-recurrence risk group. All patients initially had localized disease and were treated with radical radiotherapy with or without ADT. In this regard, two ML algorithms, artificial neural network and naïve Bayes, were examined. To the best of our knowledge, this is the first study to develop a postirradiation recurrence prediction model using ML algorithms in PC carcinoma patients. This approach was previously used in order to predict the three- and five-year biochemical recurrence in PC patients initially treated surgically [27]. Also, one of the previous studies developed a model for the prediction of the presence of bone metastases, but this study had mixed early-stage cases with those who had advanced stage of the disease, and also both newly diagnosed patients and the ones who were previously treated [28]. The advantage of our homogenous cohort (only initially localized and only radiotherapy treated) lies in the potential to apply the obtained results in daily clinical practice in this specific setting.

This study included 109 patients with PC, all treated by definitive radiotherapy with or without ADT. We tested two different ML algorithms, ANN and NB, in terms of their potential to predict disease progression in PC patients. A comparison of ML models identified that the NB model gave a better performance. This ML-based model may potentially be applicable in clinical practice when disease progression is questionable during follow-up. ML-based assistance would have a great advantage towards the prediction of the disease progression and would aid physicians to shape the treatment plan.

The important pretreatment variables for PC patients are T stage, GS, and initial PSA level. Using univariate analysis, it was found that GS is significantly associated with the occurrence of PC recurrence, whereas there was no significant difference between T stage and disease progression although a positive trend was found. Early prediction of PC recurrence based on initial characteristics of the disease has inspired multiple modeling approaches, including nomograms [29–33]. D'Amico's risk classification first suggested a risk stratification system based on three-groups in order to predict a 2-year PSA failure rate following radical prostatectomy or EBRT. It categorizes patients into low, intermediate, and high risk groups based on their initial PSA, clinical stage, and biopsy GS [29, 34]. In order to develop an algorithm to predict recurrence in PC patients treated with radical radiotherapy, Gabriele et al. carried out a Candiolo nomogram that separated patients according to five risk groups that incorporated age, pretreatment PSA, clinical-radiological staging, GS, and the percentage of positive cores from the biopsy. The Candiolo nomogram appeared to be better and capable of predicting PC recurrence following a patient's radiotherapy than the traditional D'Amico risk classes [33]. An external validation study of this nomogram which was recently conducted also indicated that clinical use of Candiolo nomograms could be justified in PC patients prior to receiving radical radiotherapy [34].

Our study also looked into the impact of posttreatment characteristics on disease progression through examining nadir PSA, time to nadir PSA, PSADT, PSA velocity, and PSA in the moment of disease reevaluation. In order to predict possible PC recurrence from the given dataset, we calculated posttreatment cut-off values for all variables. Nadir PSA and time to nadir PSA were found to be significant prognostic factors in our univariate analyses. Literature cut-off values for nadir PSA varied between 0.1 and 0.7 ng/mL when nadir PSA was used as a continuous or dichotomized variable [35–37]. The cut-off value in our study was 0.3095 ng/mL, which might be attributable to the study's inclusion criteria in which only patients who experienced a PSA increase were selected and those who did not have any increase were omitted. In our univariate analysis, PSADT and PSA velocity was also associated with disease progression. A cut-off value of 5 months in our study was found to have the strongest association with disease recurrence. Among the studies which investigated the PSADT, different cut-off values were proposed and there was no agreement about one, singular cut-off value that could be identifiable as most significant for disease recurrence. Nevertheless, a majority of studies reported a PSADT cut-off of <12 months being associated with an increased risk of disease recurrence, which does come in accordance with the result yielded in our study [38].

The majority of patients included in our study had disease recurrence localized in the bones (42%). BS was used as a primary diagnostic imaging tool, which is able to evaluate a patient's whole body and may indicate the need for additional imaging when bone metastases are suspected [39]. Though the diagnostic sensitivity of BS is generally considered to be satisfactory, it is limited in terms of specificity due to the tracer's uptake in benign processes [40]. BS may not be feasible in lower PSA levels, particularly in cases of biochemical recurrence and early localized bone marrow metastases. However, in such instances, PET/CT may be used to examine disease progression as it may yield higher accuracy than does conventional imaging [41]. Choline PET/CT, fluciclovine PET/CT, and PSMA PET/CT were the most frequently utilized in PC recurrence prediction. The diagnostic performance of BS and these PET/CT tracers has been compared within the literature. Despite its superior specificity in comparison to BS (98–100%), Picchio et al. concluded that choline PET/CT had low sensitivity [42]. Chen et al. reported that fluciclovine-PET/CT detected more bone metastases than did BS. Furthermore, there were no BS-identified lesions that were not found by fluciclovine PET/CT [43]. Recurrent PC is currently the most common indication for the use of PSMA PET/CT, which the majority of the literature is dedicated to [44]. Lengana et al. demonstrated PSMA PET/CT to have a higher sensitivity and accuracy compared to BS, including also detection of lytic as well as bone marrow metastases [44]. Moreover, a recent meta-analysis conducted by Wang et al. found PSMA PET/CT to have a significantly higher detection rate than 18F-choline and 18F-fluciclovine, particularly for low PSA levels (i.e., rates of 18F-labeled choline, fluciclovine, and PSMA were 35, 23, and 58%, respectively, for a PSA level lower than 0.5 ng/mL) [45]. Therefore, we aimed to create an algorithm based on daily practice that could guide physicians to perform more advanced imaging modalities in patients who have unfavorable prognostic features especially when the standard imaging evaluation is negative.

Zlotta et al. used the artificial neural network in order to predict the pathological stage before radical prostatectomy based on clinical, biochemical, and biopsy data and achieved classification accuracy of more than 90% [46]. Other research conducted by Wang et al. used the ANN in order to predict patients' outcomes after radical retropubic prostatectomy (RRP) on the basis of GS, surgical margins status, and organ confinement status using biochemical recurrence or nonrecurrence as output variables [47]. In our study, NB achieved the sensitivity of 100% and specificity of 92.3%. A similar study conducted by Chiu et al. achieved to build an ANN model of predicting bone metastases in prostate carcinoma patients using PSA levels and patients' age like inputs variables with good sensitivity (87.5%) and specificity (83.3%) [28]. The strength of our work lies in the fact that we included only patients with initially localized disease treated with radical radiotherapy.

There are a couple of similar studies which try to develop artificial intelligence predictive models in some other carcinomas [48–51]. A recent study conducted by Alongi et al. aimed to implement ML models in the area of choline PET/CT images in the moment when PC recurrence was suspected. They achieved to develop a model which can be implemented in clinical practice in order to select choline PET/CT features predictive of disease progression in PC patients [52]. Our main finding was that artificial intelligence models based on pretreatment and posttreatment parameters could be implemented with good accuracy in the prediction of disease progression in patients with PC initially treated with radical radiotherapy with or without ADT.

The limitations of our study include the retrospective approach and the fact that this was a single-institution analysis. All the patients included in our study had some increase of PSA level during follow-up period, which influenced the obtained cut-off values and their significance. These results provide a basis for further validation studies in larger prospective cohorts of patients as well as meta-analyses which might point to population-specific factors important in this setting.

## 5. Conclusions

In this study, ML classifiers have shown potential for application in routine clinical practice during the follow-up of PC patients when disease progression was suspected. Earlier detection of disease progression could be crucial for patients' classified into high-risk group where earlier introduction of second-line therapy can be beneficial. Also, when standard workup is negative and the classifier indicates that there is a high likelihood for disease progression, metabolic imaging could be introduced followed by active surveillance or further treatment when necessary.

## Figures and Tables

**Table 1:** Comparison of patients', disease, treatment, and follow-up characteristics between two groups of patients.

Characteristics	N (%)		Wilcoxon rank sum test
	Group 1	Group 2	
Age (years)			
Mean (SD)	70.9 (6.0)	71.3 (5.8)	*p* = 0.729
Median (range)	71.0 (55.0-84.0)	71.5 (60.0-80.0)	
T in clinical TNM			
T1	1 (1.3%)	0 (0%)	*p* ^∗^ = 0.0001
T2	70 (88.6%)	16 (23.3%)	
T3	8 (10.1%)	13 (43.3%)	
T4	0 (0%)	1 (3.3%)	
N in clinical TNM			
N0	75 (94.9%)	29 (96.7)	*p* ^∗^ = 1
N1	4 (5.1%)	1 (3.3)	
UICC^2^ staging			
I	17 (21.5%)	4 (13.3%)	*p* ^∗^ = 0.0003
II	53 (67.1%)	12 (40.0%)	
III	6 (7.6%)	13 (43.3%)	
IV	3 (3.8%)	1 (3.3%)	
Baseline PSA^1^ (ng/mL)			
Mean (SD)	48.0 (49.8)	44.4 (57.3)	*p* = 0.709
Median (range)	30.1 (4.5-249.4)	30.8 (7.0-311.0)	
Gleason's score (category)			
Low risk (GS^3^ 6)	8 (10.1%)	6 (20.0%)	*p* ^#^ = 0.317
Intermediate risk (GS^3^ 7)	42 (53.2%)	16 (53.3%)	
High risk (GS^3^ 8-10)	29 (36.7%)	8 (26.7%)	
ADT^4^			
No	23 (29.1%)	12 (40.0%)	*p* ^#^ = 0.277
Yes	56 (70.9%)	18 (60.0%)	
ADT^4^ duration (months)			
Mean (SD)	12.4 (2.6)	12.8 (1.4)	*p* = 0.15
Median (range)	12.0 (3.0-24.0)	12.0 (12.0-15.0)	
Radiotherapy (Gy)			
Mean (SD)	66.6 (2.7)	66.4 (2.4)	*p* = 0.741
Median (range)	65 (65-72)	65 (65-72)	
Nadir PSA^1^ (ng/mL)			
Mean (SD)	2.0 (5.6)	4.0 (9.9)	*p* = 0.376
Median (range)	0.4 (0-42.9)	0.6 (0-49.9)	
Time to nadir PSA^1^ (months)			
Mean (SD)	7.2 (5.5)	5.9 (4.6)	*p* = 0.176
Median (range)	6.0 (1.0-31.0)	4.0 (1.0-21.0)	
PSA^1^ doubling (months)			
Mean (SD)	6.9 (8.4)	7.6 (9.9)	*p* = 0.992
Median (range)	3.9 (0.8-50.0)	3.7 (0.9-42.0)	
PSA^1^ velocity (ng/mL/month)			
Mean (SD)	2.2 (4.2)	2.4 (3.9)	*p* = 0.762
Median (range)	0.6 (0-26.1)	1.0 (0-15.4)	
PSA^1^ in the moment of disease reevaluation			
Mean (SD)	23.1 (33.5)	19.5 (24.8)	*p* = 0.642
Median (range)	12.3 (0.3-200.4)	12.5 (0.04-120.0)	
Disease progression			
No	25 (31.6%)	12 (40.0%)	*p* ^#^ = 0.411
Yes	54 (68.4%)	18 (60.0%)	
Site			
Local recidive	8 (10.1%)	1 (3.3%)	*p* ^∗^ = 0.439
Regional lymph nodes	7 (8.9%)	3 (10.0%)	*p* ^∗^ = 1
Distant lymph nodes	4 (5.1%)	2 (6.7%)	*p* ^∗^ = 0.666
Bone	34 (43.0%)	12 (40.0%)	*p* ^#^ = 0.774
Liver	1 (1.3%)	0 (0%)	*p* ^∗^ = 1
Lung	2 (2.5%)	0 (0%)	*p* ^∗^ = 1
Biochemical relapse	11 (13.9%)	4 (13.3%)	*p* ^∗^ = 1
No. pts	79 (100%)	30 (100%)	

^1^PSA = prostate-specific antigen; ^2^UICC = Union for International Cancer Control; ^3^GS = Gleason's score; ^4^ADT = androgen deprivation therapy; ^∗^Fisher exact test; ^#^Pearson *χ*2 test.

**Table 2:** Comparison of characteristics among patients with and without disease progression in the training group.

Characteristic	The presence/absence of disease progression - training group		
	Presence	Absence	Wilcoxon rank sum test
Age (years)			
Mean (SD)	70.8 (6.3)	71.2 (5.3)	*p* = 0.987
Median (range)	71.0 (55.0-84.0)	72.0 (60.0-84.0)	
Baseline PSA			
Mean (SD)	53.2 (53.2)	36.8 (40.1)	*p* = 0.124
Median (range)	35.8 (4.5-249.0)	27.0 (5.6-190.0)	
T in clinical TNM			
T1 and T2	8 (14.8%)	0 (0%)	*p* ^#^ = 0.05
T3 and T4	46 (85.2%)	25 (100%)	
N in clinical TNM			
N0	50 (92.6%)	25 (100%)	*p* ^#^ = 0.301
N1	4 (7.4%)	0 (0%)	
UICC^1^ staging			
I and II	45 (83.3%)	25 (100%)	*p* ^#^ = 0.05
III and IV	9 (16.7%)	0 (0%)	
Gleason's score (category)			
Low risk (GS^2^ 6)	4 (7.4%)	4 (16.0%)	*p* ^#^ = 0.004
Intermediate risk (GS^2^ 7)	24 (44.4%)	18 (72.0%)	
High risk (GS^2^ 8-10)	26 (48.2%)	3 (12.0%)	
*Nadir PSA^3^*			
Mean (SD)	2.7 (6.6)	0.6 (1.0)	*p* = 0.04
Median (range)	0.7 (0-43.0)	0.1 (0-4.2)	
Time to nadir PSA^3^			
Mean (SD)	5.7 (3.4)	10.6 (7.4)	*p* = 0.0005
Median (range)	5.0 (1.0-15.0)	8.0 (3.0-31.0)	
PSA^3^ doubling time			
Mean (SD)	3.6 (2.4)	14.0 (11.8)	*p* ≤ 0.01
Median (range)	3.1 (0.8-12.9)	11.5 (2.1-50.0)	
PSA^3^ velocity			
Mean (SD)	3.1 (4.9)	0.2 (0.2)	
Median (range)	1.4 (0.1-26.1)	0.1 (0-0.8)	*p* ≤ 0.01
PSA^3^ in the moment of disease reevaluation			
Mean (SD)	32.1 (37.2)	3.6 (3.1)	*p* ≤ 0.01
Median (range)	19.1 (3.9-200.4)	3.1 (0.3-15.5)	
Total	54 (68.4%)	25 (31.6%)	*—*

^1^UICC: Union for International Cancer Control; ^2^GS: Gleason's score; ^3^PSA: prostate-specific antigen; ^#^Fisher exact test.

**Table 3:** Results of the ROC analysis for nadir PSA, time to nadir PSA, PSA doubling time, PSA velocity, PSA in the moment of disease reevaluation, and relevant events.

Characteristics	Nadir PSA	Time to nadir PSA	PSA doubling time	PSA velocity	PSA in the moment of disease reevaluation
AUC ROC^a^ (95% CI)	64.3% (51.7-76.9%)	74.2% (62.6-85.8%)	87.2% (78.8-95.5%)	91.8% (86.0-97.6%)	97.1% (93.3-100%)
Likelihood ratio test^b^	*p* < 0.01	*p* < 0.01	*p* < 0.01	*p* < 0.01	*p* < 0.01
ROC cut-off value^c^	0.3095	6.5	5.05	0.55	6.49
Sensitivity (95% CI)	63.0% (50.0-75.9%)	66.7% (53.7-77.8%)	79.6% (68.5-90.7%)	72.2% (59.2-83.3%)	94.4% (89.0-100%)
Specificity (95% CI)	68.0% (48.0-84.0%)	80.0% (64.0-96.0%)	84.0% (68.0-96.0%)	92.0% (80.0-100%)	92.0% (80.0-100%)

^a^Area under the ROC curve (DeLong's method); ^b^Likelihood ratio test for AUC ROC; ^c^Value with maximum sensitivity and specificity.

**Table 4:** The value of nadir PSA, time to nadir PSA, PSA doubling time, PSA velocity, and PSA in the moment of disease reevaluation, in prediction of disease progression.

Characteristic	The presence/absence of disease progression – training group		
	Presence	Absence	Wilcoxon rank sum test
Nadir PSA (ROC cut-off value)			
≤0.3095	20 (37.0%)	17 (68.0%)	*p* = 0.01
>0.3095	34 (63.0%)	8 (32.0%)	
Time to nadir PSA (ROC cut-off value)			
≤6.5	36 (66.7%)	5 (20.0%)	*p* = 0.0001
>6.5	18 (33.3%)	20 (80.0%)	
PSA doubling time (ROC cut-off value)			
≤5.05	43 (79.6%)	4 (16.0%)	*p* < 0.01
>5.05	11 (20.4%)	21 (84.0%)	
PSA velocity (ROC cut-off value)			
≤0.55	15 (27.8%)	23 (92.0%)	*p* < 0.01
>0.55	39 (72.2%)	2 (8.0%)	
PSA in the moment of disease reevaluation (ROC cut-off value)			
≤6.49	3 (5.6%)	23 (92.0%)	*p* < 0.01
>6.49	51 (94.4%)	2 (8.0%)	
Total	54 (68.4%)	25 (31.6%)	*—*

**Table 5:** Sensitivity, specificity, positive predictive value, negative predictive value and predictive accuracy for ANN and NB models for prediction of disease progression, and comparison between models.

Characteristics	ANN	NB	DeLong's test^∗^
AUC (95% CI)	91.7% (76.2-100%)	97.7% (92.9-100%)	Z = −0.7718, *p* = 0.4402
Likelihood ratio test^b^	*p* < 0.01	*p* < 0.01	
ROC cut-off value^c^	0.766	0.983	—
Sensitivity (95% CI)	94.4% (72.7-99.9%)	100.0% (80.5-100.0%)	
Specificity (95% CI)	91.7% (61.5-99.8%)	92.3% (64.0-99.8%)	
PPV (95% CI)	94.4% (72.2-99.1%)	94.4% (72.1-99.1%)	
NPV (95% CI)	91.7% (61.9-98.7%)	100%	
Predictive accuracy (95% CI)	93.3% (77.9-99.2%)	96.7% (82.8-99.9%)	

AUC: area under the curve; CI: confidence interval; NPV: negative predictive value; PPV: positive predictive value; ^∗^DeLong's test for two correlated ROC curves.

**Table 6:** Importance of inputs.

	Input 1	Input 2	Input 3	Input 4	Input 5	Input 6	Input 7
	UICC^1^ stage	GS^2^ risk group	Nadir PSA^3^	Time to nadir PSA^3^	PSA^3^ doubling time	PSA^3^ velocity	Follow-up PSA^3^
Relative importance (%)	26.2	13.4	9.4	8.8	9.7	11.7	20.7

^1^UICC: Union for International Cancer Control; ^2^GS: Gleason's score; ^3^PSA: prostate-specific antigen.

## Data Availability

The datasets used during the current study are available from the corresponding author on reasonable request.
